# Calling a Truce: Enterococcus faecalis as a mediator between E. coli and C. albicans during polymicrobial CAUTIs

**DOI:** 10.21203/rs.3.rs-9545567/v1

**Published:** 2026-05-07

**Authors:** Ana Flores-Mireles, Elsa Wongso, Jonathan Molina, Alex Gibbon

**Affiliations:** University of Notre Dame; University of Notre Dame; University of Notre Dame; University of Notre Dame

**Keywords:** E. faecalis, E. coli, C. albicans, CAUTI, UTI, filamentation, hyphae, biofilm, urine, bladder, catheter, bacteria, fungi, competition, cooperation, inhibition, antibiotic

## Abstract

Polymicrobial catheter-associated urinary tract infections (CAUTIs) are a severe clinical burden. CAUTIs promote colonization by different microbes, including E. faecalis, E. coli, and C. albicans. These mixed biofilms can compromise antimicrobial efficacy and promote resistance. Despite their prevalence, the cross-kingdom dynamics driving these infections within the catheterized bladder remain poorly understood. Using established in vitro and in vivo CAUTI models, we elucidate the complex, environment-dependent interactions governing these multispecies biofilms. We demonstrate that E. coli strongly antagonizes C. albicans, by limiting iron uptake, inhibiting filamentation, and inducing cell death. Crucially, we identify E. faecalis as a protective keystone species that mitigates this antagonism. E. faecalis facilitates multispecies coexistence by acting as a spatial bridge on the urothelium, enabling E. coli and C. albicans to co-localize rather than occupy segregated niches. These mechanistic insights into polymicrobial synergy and competition provide a crucial framework for developing targeted therapeutics against recalcitrant CAUTIs.

## Teaser

Dissecting the interactions between E. faecalis, E. coli, and/or C. albicans duringpolymicrobial infections in CAUTI.

## INTRODUCTION

Polymicrobial biofilms drive persistent infections across diverse body sites by shielding communities from antimicrobials^1–3^. On urinary catheters, these biofilms are the primary catalyst for catheter-associated urinary tract infections (CAUTIs)^4^, which account for ~80% of all complicated UTIs^5^. Unlike uncomplicated UTIs, which predominantly affect women and are driven by uropathogenic *Escherichia coli* (UPEC), CAUTIs exhibit no gender preference and are caused by a cross-kingdom array of pathogens^6–9^. Among these are UPEC(23.9%), *Enterococcus spp*.(13.8%)*, Candida spp*. (17.8%), *Pseudomonas aeruginosa* (10.3%), and *Klebsiella pneumoniae* (10.1%)^6,10^. While historically viewed as monomicrobial, recent clinical data reveal that up to 77% of CAUTIs, and up to 97% in long-term catheterized patients, are polymicrobial^11–13^.

CAUTIs represent a severe clinical burden, often a leading cause of secondary nosocomial bloodstream infections and urosepsis^14–19^. Complicated UTIs drive ~25% of all sepsis cases^20^, with mortality rates rising from 10%^19,21,22^ to 63% during polymicrobial bacteremia^23–26^. Furthermore, the high prevalence of multi-drug resistant uropathogen strains, including ESKAPE pathogens, underscores the urgent need to prevent both monomicrobial and polymicrobial CAUTIs^27–30^.

The three most prevalent early colonizers in CAUTI are the gram-negative *E. coli*, the gram-positive *Enterococcus* spp.and the fungal pathogen *Candida* spp.^6,10,31–33^. While *E. faecalis* and *E. coli* are frequently co-isolated^8,34–37^, clinical studies increasingly note their co-occurrence with *C. albicans*^38–42^. Despite this, the polymicrobial dynamics and pathogenesis of *C. albicans* within the bladder remain poorly understood^6,43^.

In this study, we utilized *in vitro* and *in vivo* murine CAUTI models to investigate the complex interplay between *E. faecalis*, *E. coli*, and *C. albicans*. We demonstrate that while *E. coli* dominates competitively *in vitro* under nutrient limitation, this advantage is attenuated *in vivo*. Mechanistically, *E. coli* secretes small proteins that suppress C. albicans growth and hyphal formation, a phenotype linked to the transcriptional downregulation of fungal iron and metal uptake pathways. Crucially, *E. faecalis* acts as a protective mediator, abrogating this fungal inhibition. Furthermore, *E. faecalis* functions as a spatial bridge on the bladder urothelium, enabling the co-localization of *E. coli* and *C. albicans*, which otherwise occupy segregated niches. Uncovering these specific antagonistic (metal sequestration) and synergistic (*E. faecalis* mediation) interactions provides vital mechanistic insights for future development of targeted therapeutics against recalcitrant polymicrobial biofilms.

## RESULTS

### *C. albicans* biofilm formation and survival is inhibited by *E. coli* in *in vitro* urine conditions.

**Figure 1:**
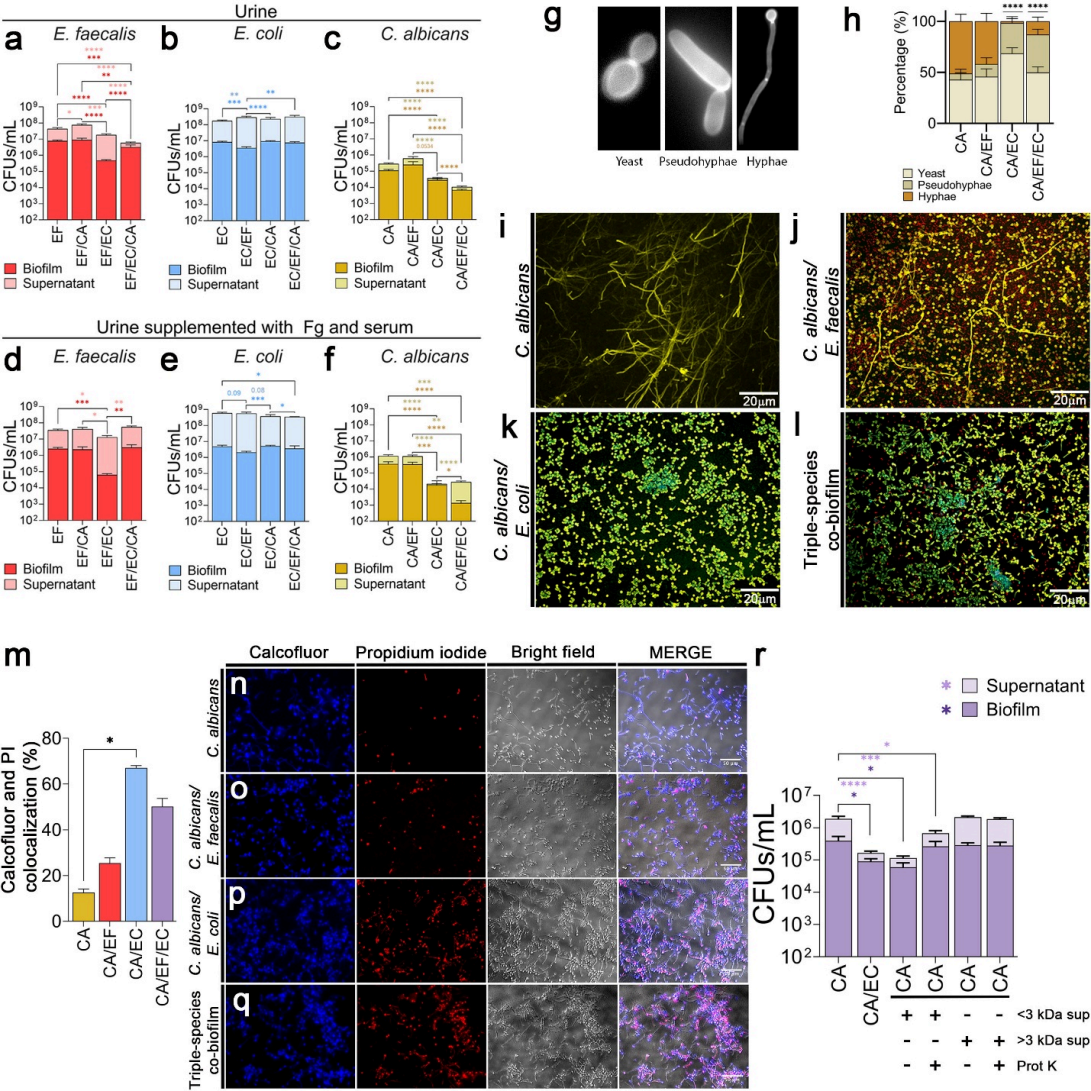
*E. coli* inhibits the growth and hyphal formation of *C. albicans* through small, secreted proteins (<3 kDa). **(a-f)** Biofilms of *E. faecalis* (EF), *E. coli* (EC), and *C. albicans* (CA) grown for 48 hours in human urine and urine + Fg (3 mg/mL) + Serum (10%). Biofilms were grown on silicone disks covered with 1 mg/mL Fg overnight. Red/pink columns show EF biofilm/supernatant with EC (EF/EC), CA (EF/CA), or both (EF/EC/CA). Blue/light blue graphs show EC biofilm/supernatant with EF (EC/EF), CA (EC/CA), or both (EC/EF/CA). Yellow, light yellow columns show CA biofilm/supernatant with EF (CA/EF), EC (CA/EC), or both (CA/EF/EC). **(g)** Reference for *C. albicans* morphologies **(h)** Percentage of yeast, pseudohyphae, and hyphae in different co-biofilms. Chi-squared test was used to identify significance in morphology distribution between *C. albicans* monoculture and during polymicrobial interaction (****, *p* < 0.0001). **(i-l)** Biofilms were grown in human urine for 48 hours on silicone sheets (cut into 1”×1” square) that was covered with Fg (1 mg/mL) overnight. Biofilms were stained using antibody against Fg (anti-Fg; green), *Enterococcus* (anti-EbpABC; red), *E. coli* (anti-*E. coli;* turquoise), and *C. albicans* (anti-*Candida;* yellow). Coverslips were mounted on the silicone sheets and imaged at 20× magnifications. **(m)** Co-localization of propidium iodide (PI) and calcofluor white stains on biofilms. **(n-q)** Biofilms were grown on 96-well plates covered with Fg overnight and incubated with urine for 24 hours. Biofilms were fixed with 10% formalin and stained with PI and calcofluor white and image at 40× magnifications. Images were imaged using Zeiss inverted light microscope. **(r)** *E. coli* was grown in urine for 48 hours and filter sterilized. Supernatants were separated into >3 kDa and <3 kDa. Small proteins were further concentrated using vacufuge. Supernatants were used to grow *C. albicans* biofilms for 48 hours and CFUs were measured. Differences between groups were tested for significance using the Mann-Whitney U test. *, *p* < 0.05; **, *p* < 0.005; ***, *p* < 0.0005; and ****, *p* < 0.0001.

Catheter-induced tissue damage results in edema and plasma protein extravasation into the bladder, resulting in accumulation of fibrinogen (Fg), the key host clotting factor^6,8,44–51^. *E. coli, E. faecalis*, and *C. albicans* exploit this deposited Fg to form persistent biofilms on bladders and catheters^35–38^. To mimic this environment, we utilized an *in vitro* Fg-urine assay to compare single-, dual-, and triple-species biofilm dynamics. Cultures were grown for 48 hours on Fg-coated silicone disks in filter-sterilized pooled human urine, which was supplemented with Fg and/or human serum to simulate extravasation^47,48,52,53^ (Fig. 1d–f, **Extended Data Fig. 1**). We quantified colony-forming units (CFUs) in both the biofilm and planktonic (supernatant) fractions to assess overall population levels and specific interspecies competition (Fig. 1a–f, **Extended Data Fig. 1g-l**).

*E. faecalis* (total populations) did not compete significantly with *C. albicans*, but its overall abundance was significantly reduced by *E. coli* (**Extended Data Fig. 1a,d,m,p**). This reduction was biofilm-specific alongside increased planktonic growth (Fig. 1a,d), indicating surface displacement by *E. coli*. In triple-species cultures, *E. faecalis* biofilm and planktonic growth decreased in non-supplemented urine, but this was rescued by Fg/serum supplementation, primarily within the biofilm (Fig. 1a,d, **Extended Data Fig. 1a,d,g,j,m,p**), suggesting nutritional competition.

Overall, *E. coli* maintained stable populations across most interactions (**Extended Data Fig. 1b,e,h,k**). Only two significant shifts occurred: First, total *E. coli* increased during *E. faecalis* co-culture in non-supplemented urine (**Extended Data Fig. 1b**), shifting from biofilm to planktonic population (Fig. 1b). Second, total *E. coli* decreased during triple-species competition in serum-supplemented urine due to a planktonic population drop (**Extended Data Fig. 1k,q**). Despite Fg and serum supplementation, this competitive dynamic persisted, suggesting that *E. coli* and *E. faecalis* compete primarily for spatial adherence, not nutrients (Fig. 1e, **Extended Data Fig. 1e**).

*C. albicans* was the most severely impacted microbe. While *E. faecalis* exhibited no antagonism and even enhanced fungal populations in Fg-urine(Fig. 1c,f, and **Extended Data Fig. 1c,f,i,l**), *E. coli* consistently reduced both biofilm and planktonic fungal populations in all conditions, regardless of supplementation (Fig. 1c,f, and **Extended Data Fig. 1o,r**). Strikingly, in Fg/serum-supplemented triple cultures, total fungal populations significantly increased (**Extended Data Fig. 1f**). Interestingly, *E. faecalis* shifted fungal biomass toward the planktonic population, effectively rescuing *C. albicans* from *E. coli*-mediated inhibition (Fig. 1f, and **Extended Data Fig. 1f**).

Because standard CFUs underestimate multinucleated hyphae^39,54^, we used the ITS2-targeted qPCR^39^ to distinguish true growth inhibition from filamentation (hyphal formation) (**Extended Data Fig. 2a-b**). In planktonic populations, qPCR counts were significantly higher than CFUs during both dual- and triple-species interactions (**Extended Data Fig. 2a**). In biofilms, however, qPCR counts significantly exceeded CFUs only in the triple-species model (**Extended Data Fig. 2b**). This discrepancy suggests that the simultaneous presence of *E. faecalis* and *E. coli* uniquely drives robust hyphal formation within the biofilm.

Together, these results reveal that the presence of both *E. coli* and *E. faecalis* uniquely drives robust biofilm hyphal formation. Ultimately, while *E. coli* is competitively dominant and heavily suppresses *C. albicans*, *E. faecalis* mitigates this antagonism in triple-species environments.

### *E. coli*’ssecreted factors inhibit *C. albicans* viability

To determine whether these interactions are contact-dependent, we incubated *C. albicans* with concentrated cell-free supernatants from *E. faecalis*, *E. coli*, or their co-culture in urine for 24 and 48 hours. *E. coli* supernatant significantly inhibited fungal biofilms, whereas *E. faecalis* and mixed-culture supernatants did not (**Extended Data Fig. 3a**). Partitioned chamber assays confirmed that *E. coli* suppresses *C. albicans* via contact-independent factors (**Extended Data Fig. 3b,c**). Furthermore, temporal planktonic growth assays revealed that *E. coli* consistently suppresses the fungus regardless of urine supplementation (**Extended Data Fig. 4a**) without suffering reciprocal growth defects (**Extended Data Fig. 4b**). Together, these results demonstrate that *E. coli* antagonizes *C. albicans* via secreted factors, an effect efficiently neutralized by soluble factors from *E. faecalis*.

### *E. coli* inhibits fungal hyphal formation and damages fungal membrane in *C. albicans* biofilms.

Because our qPCR data indicated that polymicrobial interactions alter fungal filamentation (**Extended Data Fig. 2a-b**), we investigated how these environments influence *C. albicans* morphology, specifically the transition from yeast to pseudohyphae and hyphae (Fig. 1g), a crucial virulence determinant for tissue invasion and biofilm formation essential for CAUTI progression^49,55–59^. Immunofluorescence microscopy of biofilms grown on Fg-coated plates in urine (Fig. 1g–l) revealed that *C. albicans* monocultures formed robust hyphae representing ~50% of the population (Fig. 1h,i), corroborating our previous findings^49^. While *E. faecalis* caused only a modest reduction in filamentation (Fig. 1h,j), *E. coli* nearly eradicated hyphal formation (Fig. 1h,k). Strikingly, *E. faecalis* rescued fungal morphology in triple-species biofilms, significantly increasing pseudohyphal proportions (Fig. 1h,l).

To distinguish between growth inhibition and active killing, we assessed viability in 24-hour Fg-urine biofilms (Fig. 1m–q). Fungal cells were stained with calcofluor white^60,61^ and propidium iodide (PI) was used to detect cell death^62^. PI-positive dead fungal cells rose from ~15% in monomicrobial biofilms (Fig. 1m–n) to ~67% during *E. coli* co-culture (Fig. 1m,p). Reinforcing its protective role, *E. faecalis* mitigated this antagonism in triple-species biofilms, reducing fungal cell death by ~17% (Fig. 1m,q).

Finally, to characterize the secreted factor responsible for this inhibition, we size-fractionated cell-free supernatants from 48-hour *E. coli* Fg-urine biofilms into <3 kDa and >3 kDa fractions. We then cultured *C. albicans* biofilms in fresh human urine for 24 hours at 37°C in the presence of these fractions. To determine whether the inhibitory molecules were proteinaceous, we also subjected the fractions to Proteinase K (ProtK) treatment. The <3 kDa fraction strongly inhibited both biofilm and planktonic fungal populations, mimicking the effects of live *E. coli* co-culture (Fig. 1r). Notably, ProtK treatment of the <3 kDa fraction significantly restored fungal biofilm and planktonic populations (Fig. 1r). Conversely, the >3 kDa fraction had no effect on *C. albicans*, regardless of ProtK treatment (Fig. 1r). Together, these data demonstrate that *E. coli* secretes small (<3 kDa) candidacidal proteins that impair fungal filamentation and viability in urine.

### *E. faecalis* attenuates *E. coli*-mediated suppression of *C. albicans* pathogenesis in an *in vivo* CAUTI model.

To corroborate our *in vitro* findings, we evaluated these polymicrobial interactions using our *in vivo* murine CAUTI model. Mice were transurethrally catheterized and infected with pathogens in single-, dual-, or triple-species combinations. At 1- and 3-days post-catheterization and infection (dpci), catheters and organs (bladder, kidneys, spleen, heart) were harvested for CFU quantification. Additionally, bladders were collected for immunofluorescence and scanning electron microscopy (SEM).

**Figure 2:**
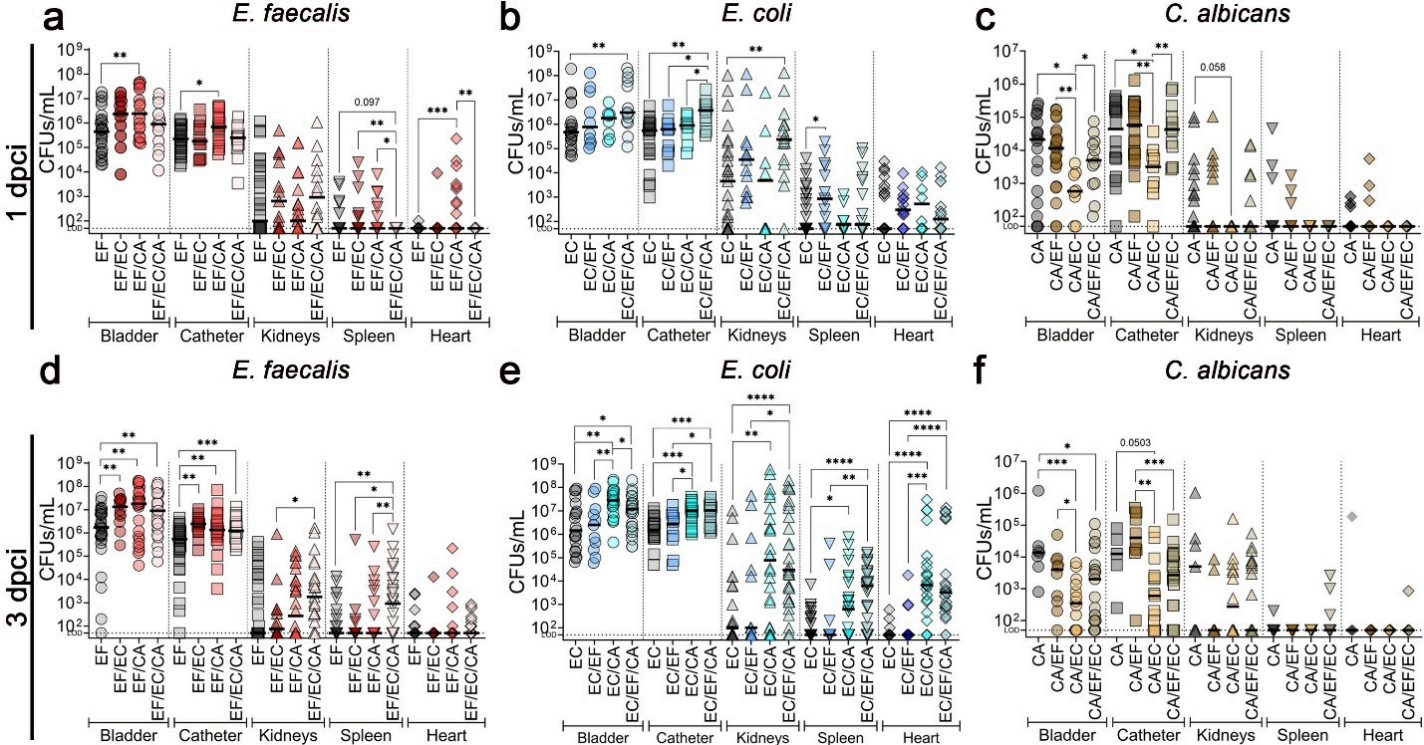
*E. faecalis* presence modulates the antagonistic interactions between *E. coli* and *C. albicans in vivo*. **(a-f)** Microbial burden of *E. faecalis* (EF)*E. coli* (EC) and *C. albicans* (CA) on the harvested organs and catheters after 1 day **(a-c)** and 3 days **(d-f)** post-catheterization and infection. **(a, d)** *E. faecalis* CFUs in co-infections with *E. coli* (EF/EC), *C. albicans* (EF/CA), and *E. coli + C. albicans* (EF/EC/CA). **(b, e)** *E. coli* CFUs in co-infections with *E. faecalis* (EC/EF), *C. albicans* (EC/CA), and *E. faecalis + C. albicans* (EC/EF/CA). **(c, f)** *C. albicans* CFUs in co-infections with *E. faecalis* (CA/EF), *E. coli* (CA/EC), and *E. faecalis + E. coli* (CA/EF/EC). Statistical analysis was done using Mann-Whitney U test (*, *p* < 0.05; **, *p* < 0.005; ***, *p* < 0.0005; and ****, *p* < 0.0001). The horizontal bar represents the median value. The horizontal broken line represents the limit of detection. LOD: limit of detection.

*E. faecalis* burdensignificantly increased in the bladder and on the catheter during co-infection with *C. albicans* at both 1- and 3-dpci, with early dissemination to the heart at 1-dpci (Fig. 2a,d). Co-infection with *E. coli* significantly enhanced enterococcal bladder burden at 3-dpci. Crucially, during triple-species infections at 3-dpci, *E. faecalis* colonization increased significantly on the catheters and across all organs except the heart (Fig. 2d).

*E. coli* colonization of the bladder and catheter also increased significantly during triple-species infections at both timepoints (Fig. 2b,e), and during co-infection with *C. albicans* at 3-dpci (Fig. 2e). While co-infection with *E. faecalis* drove significant *E. coli* dissemination to the spleen at 1-dpci (Fig. 2b), systemic *E. coli* dissemination was significantly elevated by 3-dpci during both *E. coli-C. albicans* and triple-species infections (Fig. 2e).

Consistent with our *in vitro* data, *C. albicans* colonization significantly decreased in the bladder (1- and 3-dpci) and on the catheter (1-dpci) when co-infected with *E. coli* (Fig. 2c,f). Strikingly, fungal burden in these sites rebounded, increasing significantly during both *E. faecalis* dual-infections and triple-species infections at 1-dpci (Fig. 2c). This strongly confirms that *E. faecalis* mitigates *E. coli*-mediated fungal antagonism *in vivo*.

### *E. coli* and *C. albicans* occupy distinct bladder niches, whereas *E. faecalis* promotes mixed multispecies biofilms.

**Figure 3:**
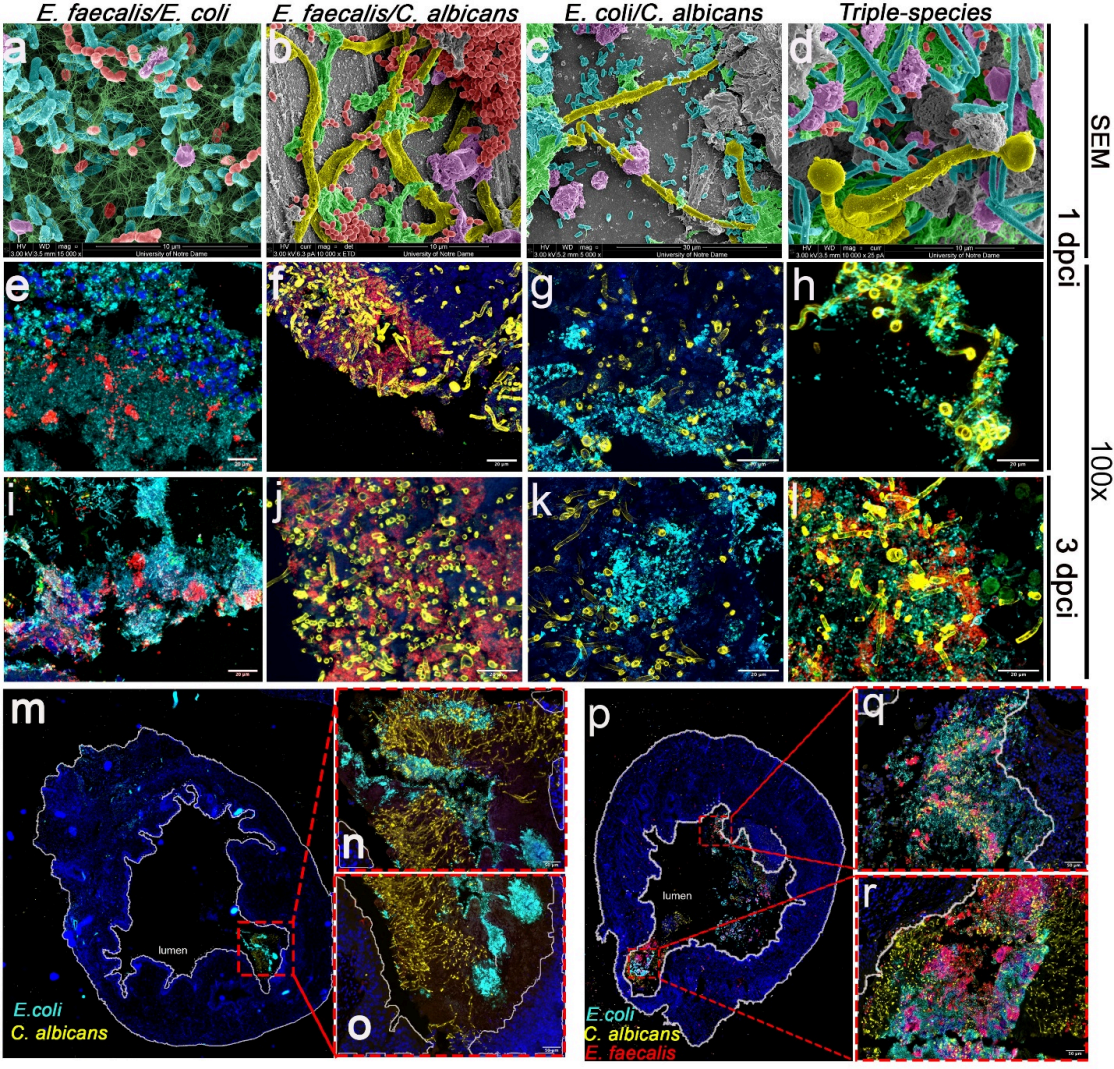
*E. coli* and *C. albicans* have limited interactions in the bladder during co-infections but form mixed biofilms in the presence of *E. faecalis*. **(a-d)** Images were taken with *The Magellan 400* - field emission scanning electron microscope (FE-SEM) and pseudo-colored using Adobe Photoshop. **(e-l)** Implanted and infected bladders were recovered and fixed at 1 dpci **(e-h)** and 3 dpci **(i-l)**. **(m)** 10× and **(n-o)** 20× images of a bladder section co-infected with *E. coli* and *C. albicans* at 3 dpci. **(p)** 10x and **(q-r)** 20× images of a bladder section co-infected with *E. faecalis, E. coli*, and *C. albicans* at 3 dpci. Bladders were subjected to analysis by IF staining or SEM. For IF analysis, antibody staining was used to detect Fg (anti-Fg; green), *E. faecalis* (anti-EbpABC; red), *E. coli* (anti-*E. coli;* turquoise), and *C. albicans* (anti-*Candida;* yellow). Staining with DAPI (blue) delineated the urothelium and cell nuclei. The white broken line separates the bladder lumen (L) from the urothelium surface (U), the lamina propria (LP), and muscularis (M). Images were taken at 100x magnifications.

To determine spatial arrangement and niche occupation during polymicrobial CAUTI, we visualized formalin-fixed bladders at 1- and 3-dpci using fluorescence microscopy and scanning electron microscopy (SEM; 1-dpci only) (Fig. 3a–l). During dual infections, *E. faecalis* readily co-localized to form mixed biofilms with both *E. coli* (Fig. 3a,e,i, **Extended Data Fig. 5a**) and *C. albicans* (Fig. 3b,f,j, **Extended Data Fig. 5b**). In sharp contrast, *E. coli* and *C. albicans* exhibited minimal co-localization, remaining segregated in distinct spatial areas across both time points (Fig. 3c,g,k,n,o, **Extended Data Fig. 5c**). Notably, during triple-species infections, all three pathogens heavily co-localized within the bladder lumen (Fig. 3d,h,l,p–r, **Extended Data Fig. 5c**). This suggests that *E. faecalis* acts as a bridge between the otherwise segregated niches of *E. coli* and *C. albicans*, facilitating the formation of complex, mixed polymicrobial biofilms.

### Established fungal biofilms exhibit resistance to *E. coli* antagonistic effects.

To investigate how colonization sequence influences polymicrobial dynamics, we established 24-hour monoculture biofilms before introducing subsequent a second or third pathogen. *E. coli* biofilm and planktonic populations remained entirely unaffected by the presence of *E. faecalis*, *C. albicans*, or both, regardless of colonization sequence (**Extended Data Fig. 6b**). However, *E. faecalis* planktonic population dropped significantly when introduced to established *C. albicans* or *E. coli* biofilms (**Extended Data Fig. 6a**).

**Figure 4:**
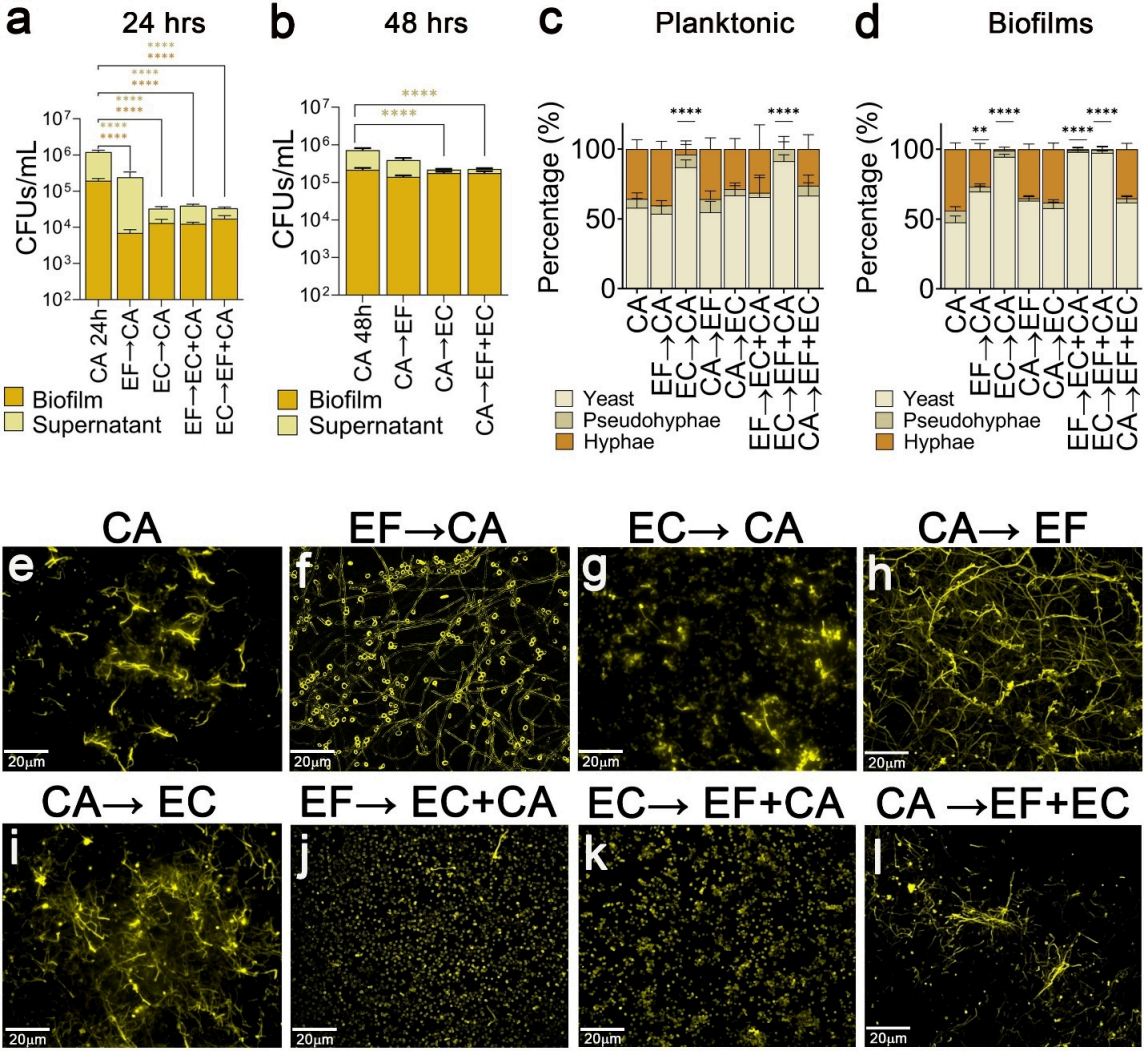
*C. albicans* mature biofilms are not affected by *E. faecalis* and/or *E. coli*. **(a, b)** *C. albicans* grown sequentially for 24 hours **(a)** or 48 hours (24 + 24 hours) **(b)** with either *E. faecalis* or *E. coli* or both. **(x→y)** x represents microorganism grown in the first 24 hours and y represents microorganism grown in the next 24 hours. Statistical analysis was done using Mann-Whitney U test (****, *p* < 0.0001). **(c, d)** The morphology of *C. albicans* in the supernatant **(c)** and biofilm **(d)**were evaluated after 48 hours of sequential growth in urine. Images (consisting of a 3 × 3 tiled region, i.e., 9 fields of view) were randomly acquired and at least three images were analyzed per condition. The total number of cells per phenotype were summed and divided by the total number of cells and the data are shown as overall percentage (%) of yeast, pseudohyphae, and hyphae. Chi-squared test was used to identify significance in morphology distribution between *C. albicans* monoculture and during polymicrobial interaction (**, *p* < 0.005; and ****, *p* < 0.0001). **(e-l)** Biofilms were grown in human urine for 48 hours (sequential) on silicone sheets (cut into 1”×1” square) that was covered with Fg (1 mg/mL) overnight. Biofilms were stained using antibody against *C. albicans* (anti-*Candida;* yellow). Coverslips were mounted on the silicone sheets and imaged using Zeiss inverted light microscope (20× magnifications).

Fungal colonization was similarly dependent on colonization order. While pre-existing bacterial biofilms significantly reduced *C. albicans* CFUs(Fig. 4a), qPCR analysis revealed elevated fungal genomic counts upon introduction to mature *E. faecalis* biofilms (**Extended Data Fig. 6c**), indicating robust hyphal formation rather than growth inhibition. Importantly, if *C. albicans* established a mature biofilm first (48 hours), the subsequent addition of *E. faecalis* and *E. coli* failed to disrupt the fungal biofilm, affecting only the planktonic population (Fig. 4b).This highlights that initial fungal colonization is critical for *C. albicans* survival during polymicrobial interactions.

Sequence-dependent survival was closely tied to fungal morphology. When *C. albicans* was inoculated onto a mature *E. faecalis* biofilm, hyphae were present in both the planktonic (Fig. 4c) and biofilm populations (Fig. 4d,e). However, when introduced to an established *E. coli* biofilm, the fungal population remained predominantly in the yeast form, with almost no hyphal development observed (Fig. 4c,d,g,k). Notably, when *C. albicans* established a mature biofilm prior to the introduction of either bacterial pathogen, it maintained a highly abundant hyphal network across both populations (Fig. 4c,d,h,i,l). Ultimately, these findings demonstrate that early establishment is crucial for *C. albicans* to survive, maintain its virulent hyphal form, and successfully resist *E. coli* antagonism.

### *C. albicans* and *E. faecalis* prior surface colonization and minimal planktonic growth overcomes *E. coli* dominance in urine

As the most prevalent uropathogen^9,37,63^, *E. coli* rapidly exploits available nutrients to grow robustly in urine, gaining a significant initial colonization advantage. To mitigate this disparity and test the importance of early establishment, we allowed a 45-minute microbial attachment phase on Fg-coated disks, washed away unbound cells (simulating urine flushing), and introduced fresh urine (with or without Fg supplementation) to assess colonization over time (**Extended Data Fig. 7a**).

During co-incubated with *E. coli*, *E. faecalis* colonization was significantly reduced only at early time points (6 and 12 hours) (**Extended Data Fig. 7b,f**), whereas co-incubation with *C. albicans* significantly enhanced early enterococcal colonization regardless of Fg (**Extended Data Fig. 7c,g**). However, in triple-species cultures, enterococcal colonization declined significantly across multiple time points (**Extended Data Fig. 7e,i**). Unsurprisingly, *E. coli* colonization was never negatively impacted; instead, its population significantly increased when co-incubated with *C. albicans* at 48 hours in non-supplemented urine, and at 24 hours with Fg supplementation (**Extended Data Fig. 7d,h**).

Fungal colonization remained unaffected by *E. faecalis* but was significantly reduced by *E. coli* at 24 hours in non-supplemented urine, an effect mitigated by Fg supplementation (**Extended Data Fig. 7d,h**). In triple-species cultures, both *E. faecalis* and *C. albicans* populations dropped significantly after 12 hours in non-supplemented urine, but this reduction was delayed to 24 hours when Fg was supplemented. This highlights how heavily nutritional stress influences surface competition. Importantly, these fungal reductions were far less drastic than those previously observed (Fig. 1–3, **Extended Data Fig. 1–4**). This confirms that initial surface binding, coupled with the removal of unattached cells (simulating urine flushing), is essential for *E. faecalis* and *C. albicans* to successfully establish biofilms in the presence of *E. coli*.

### *E. coli* downregulates *C. albicans’* iron uptake and hyphal formation genes.

**Figure 5:**
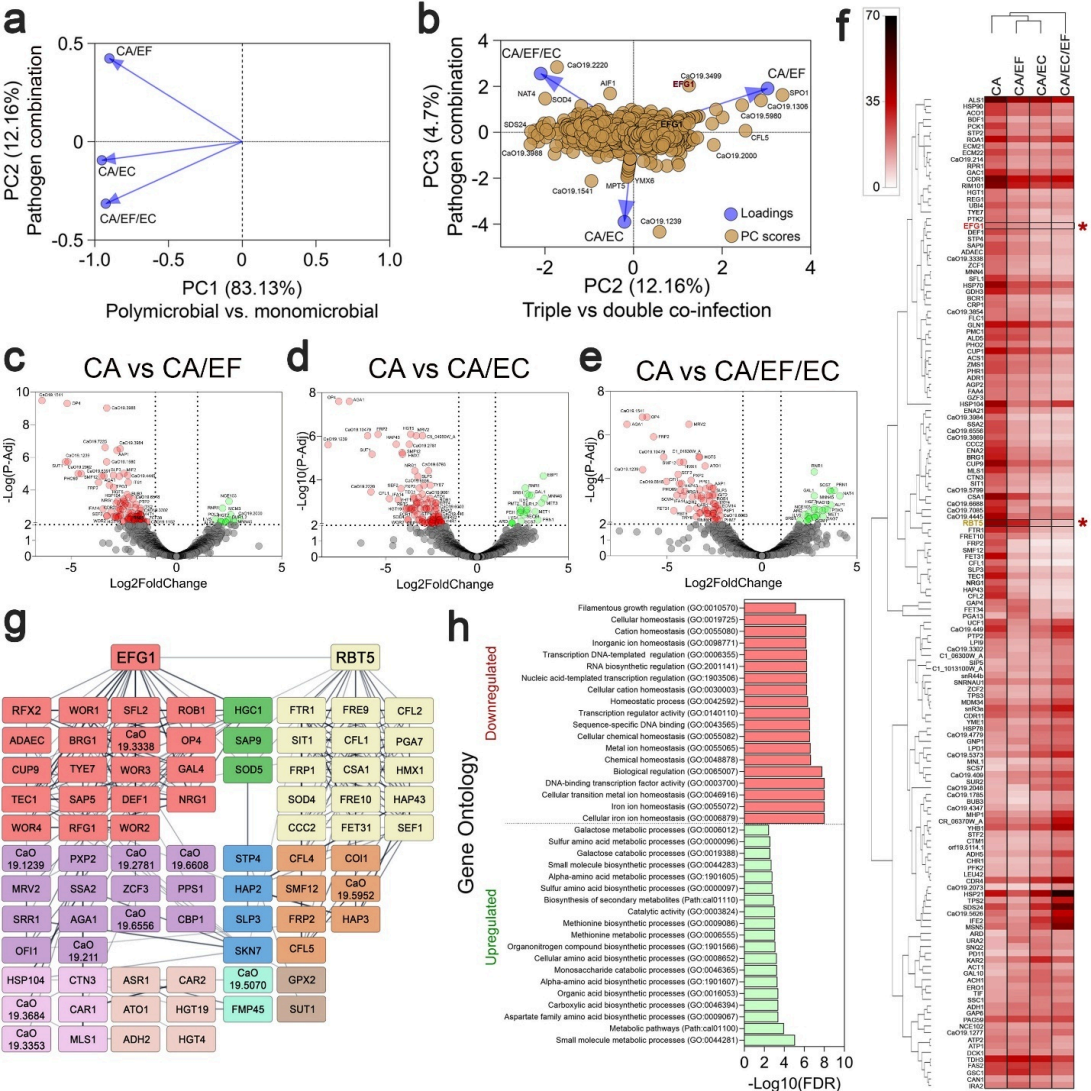
*C. albicans’* iron uptake and hyphal formation genes are downregulated in the presence of *E. coli*. **(a)** PCA plot showing different distribution of polymicrobial biofilm transcriptomes (PC1 vs. PC2). **(b)** PCA biplot showings score plots and loading plots (PC2 vs. PC3). **(c-e)** Volcano plots of differentially regulated genes of *C. albicans* during co-biofilms with *E. faecalis* (CA/EF), *E. coli* (CA/EC), or both (CA/EF/EC) in comparison with *C. albicans* biofilms. Redrepresents downregulated genes and green represents upregulated genes with a cutoff of 2 in −log(p-adj) and 1 in log2foldchange. **(f)** Top 130 *C. albicans* genes that are significantly downregulated or upregulated in the presence of *E. faecalis* (CA/EF)*, E. coli* (CA/EC), or both (CA/EF/EC). **(g)** String network analysis of downregulated genes of *C. albicans* in co-biofilms with *E. coli*. Light red represents genes that are directly related to EFG1, light yellow directly related to RBT5, green directly related to EFG1 and RBT5. Purple directly related to light red group, orangedirectly related to light yellow, blue directly related to purple and orange. **(h)** Gene ontology classifications of downregulated *C. albicans* genes in *E. coli/C. albicans* biofilms.

To elucidate the mechanisms driving these distinct polymicrobial behaviors, we performed RNA-seq on single-, dual-, and triple-species urine-Fg biofilms. Transcriptomic profiling revealed that polymicrobial environments significantly alter *C. albicans* gene expression, primarily driving widespread downregulation (Fig. 5a–e, **Extended Data Fig. 8a,b**). Notably, the expression profile of *C. albicans* monocultures closely resembled that of *C. albicans-E. faecalis* co-biofilms (**Extended Data Fig. 8e**). However, *E. coli*’s presence induced a drastic transcriptional shift that was uniquely modulated in triple-species communities (Fig. 5a,b, **Extended Data Figs. 8–10**).

Specifically, *E. coli* co-biofilms significantly downregulated fungal genes critical for filamentation (e.g., *EFG1, EED1, BRG1, NRG1, TEC1, ALS1, ROB1, SLP3, PGA7*) and iron uptake/utilization (e.g., *RBT5, SFL1, CDR1, FET34, FTR1, HAP43, FET31, FRP2*) (Fig. 5d–h). Gene Ontology (GO) analysis confirmed that cellular metal/iron ion homeostasis and filamentation regulation were the most heavily downregulated pathways during *E. coli* co-culture (Fig. 5h). Conversely, general stress response genes were broadly upregulated across all species in both dual and triple interactions (**Extended Data Fig. 9d-i, 10d-i**). Consistent with our phenotypic data, the inclusion of *E. faecalis* in these co-cultures alleviated the severe repression of fungal filamentation and iron acquisition pathways (Fig. 5f, **Extended Data Fig. 8c,d**).

The suppression of these specific pathways by *E. coli* has profound phenotypic consequences. Iron is essential for maintaining hyphal growth and overall fungal virulence^64–66^. Because the host bladder environment is inherently iron-restricted to combat infection^65,67^, *C. albicans* typically upregulates iron-acquisition genes, including HAP43, which represses iron-consuming processes while boosting uptake, under such conditions^68,69^. The paradoxical downregulation of these critical pathways in the presence of *E. coli* strongly suggests that *E. coli* produces specific factors that actively interfere with *C. albicans* iron acquisition, thereby forcing the suppression of hyphal formation and fungal growth.

While interacting with either pathogen induced stress responses in *E. faecalis*, co-biofilm with *E. coli* caused the most significant transcriptional shifts (**Extended Fig. 9**). This interaction downregulated the Ebp pilus and quorum sensing, slightly reduced the secreted proteases GelE and SprE, and strongly repressed EntV, bacteriocin, and phage-mediated lysis (LrgA-B) (**Extended Fig. 9j**). Notably, a triple interaction partially mitigated the downregulation of GelE and SprE, but EntV was further repressed.

Although *E. coli* was the least affected by polymicrobial interactions, its metabolic pathways were modulated by either pathogen (**Extended Fig. 10**). Specifically, co-culture with *E. faecalis* downregulated small toxins and type 1 pilus components (**Extended Fig. 10j**). Notably, enterobactin biosynthesis was upregulated across all interactions, peaking when *C. albicans* was present (**Extended Fig. 10j**).

## DISCUSSION

Despite technological advances, treating polymicrobial infections remains challenging due to limited therapeutics^70–72^ and standard urine cultures frequently failing to capture co-infections^73,74^. This diagnostic gap drives inadequate antibiotic use and antimicrobial resistance^75–79^, allowing infections to progress to urosepsis, which carries a significantly higher mortality rate (25%) than monomicrobial cases^11,25^. Furthermore, while bacterial co-occurrences in long-term catheterized patients are well-documented^33,41^, fungi are largely ignored. To address this, we investigated the dynamics among three common CAUTI pathogens, *E. coli*, *C. albicans*, and *E. faecalis*^6,80,81^. We found that while *E. coli* antagonizes *C. albicans*, *E. faecalis* interacts positively with the fungus *in vitro* and *in vivo*. Crucially, during triple-species infections, *E. faecalis* mitigates *E. coli*’s antagonistic effects on *C. albicans*.

This positive *E. faecalis–C. albicans* interaction in CAUTI diverges from non-urine models. In mucosal tissue models, initial synergistic invasion via upregulated fungal virulence genes has been observed^82–84^. However, other studies report this synergy shifts to antagonism by 48 hours, where *E. faecalis* downregulates critical fungal hyphal genes (*ALS1, ALS3, EFG1*)^85,86^ and secretes EntV, a bacteriocin that disrupts *C. albicans* biofilms and hyphae^39^. Conversely, our transcriptomic analysis reveals that EntV is downregulated during polymicrobial interactions in urine (**Extended Fig. 9j**). This aligns with our phenotypic finding that *E. faecalis* does not significantly impact fungal filamentation. Moreover, the master hyphal and virulence regulators, *EFG1* and *RBT5*, remained unaffected by the *E. faecalis* presence (Fig. 5). These environment-dependent dynamics highlight the critical need for urine-specific models in CAUTI research.

Previous studies report *C. albicans*-*E. coli* interactions ranging from synergistic epithelial co-invasion^87^ and antibiotic tolerance to β-lactams antibiotics^88,89^, to active antagonism via hyphal inhibition^90–93^. While an early ascending uUTI rat model suggested prior *E. coli* infection promotes fungal bladder adherence^94^; our catheterized bladder model demonstrates the opposite. *E. coli* actively kills *C. albicans* and inhibits hyphal growth (Fig. 1) by repressing *EFG1* (Fig. 5), the central virulence regulator^49,95^, forcing the fungus into a distinct spatial survival niche (Fig. 2, 3). This agrees with clinical transcriptomic data from CAUTI catheters where *C. albicans* co-colonized with *E. coli* exhibits decreased *EFG1* regulon expression compared to mono-colonized catheters^95^. This antagonism also mirrors how diverse gram-negative bacteria (e.g., *P. aeruginosa*, *A. baumannii*, *B. cepacia*, and *S. enterica*) suppress *C. albicans* via secreted molecules like quorum-sensing signals and virulence factors (e.g., phospholipase C and phenazines in *P. aeruginosa*)^96^.Similarly, our data indicate that small *E. coli*-secreted proteins mediate fungal killing and hyphal inhibition, though identifying the specific proteins responsible for these phenotypes requires further investigation.

Importantly, our data demonstrate that the sequence of colonization, known as priority effects, dictates polymicrobial composition^97^. By modifying the environment or preempting niches, initial colonizers often determine a community's future structure and exclude competitors. In our model, adding *E. faecalis* or *E. coli* to mature *C. albicans* biofilms abolishes their typical competition, indicating that early fungal attachment is critical for fungal survival (Fig. 4h,i,l). This establishment sequence also drives fungal morphology, where *C. albicans* predominantly forms hyphal biofilms if it attaches first, but remains primarily in its yeast form if *E. coli* precedes it (Fig. 4h,i,l), a morphological shift that directly aligns with clinical *EFG1* regulon repression data^95^.

Our previous studies demonstrate that fungal filamentation is critical in the catheterized bladder^49,95^, a process highly dependent on iron^64–66^. Because the host bladder is inherently iron-restricted to combat infection^65,67^, iron acquisition becomes a fierce competition. In iron-restricted conditions, *C. albicans* typically upregulates iron-acquisition genes, including *HAP43*, which boosts uptake while repressing iron-consuming processes^68,69^. However, In *C. albicans*/*E. coli* biofilms, numerous fungal iron pathways and hyphal-related genes are paradoxically downregulated. Simultaneously, *E. coli* enterobactin biosynthesis is highly upregulated (**Extended Fig. 10j**). This indicates that *E. coli* actively competes with *C. albicans* for iron via siderophores, forcing the suppression of fungal growth and filamentation. Nevertheless, additional candidacidal factors may also be at play.

Interestingly, *E. faecalis* highly upregulates two secreted proteases, SprE and GelE, during CAUTI^98^. While these proteases are known to modify the bladder environment by targeting the host fibrinolytic system to create a fibrin-rich niche ideal for biofilm persistence^51,98^, we hypothesize that they may also target and degrade the antagonistic *E. coli* small proteins. By neutralizing these *E. coli* effectors, enterococcal proteases would mitigate fungal inhibition and enable spatial co-colonization (Fig. 3). However, the role of these enterococcal proteases in degrading *E. coli* secreted proteins requires further investigation.

**Figure 6:**
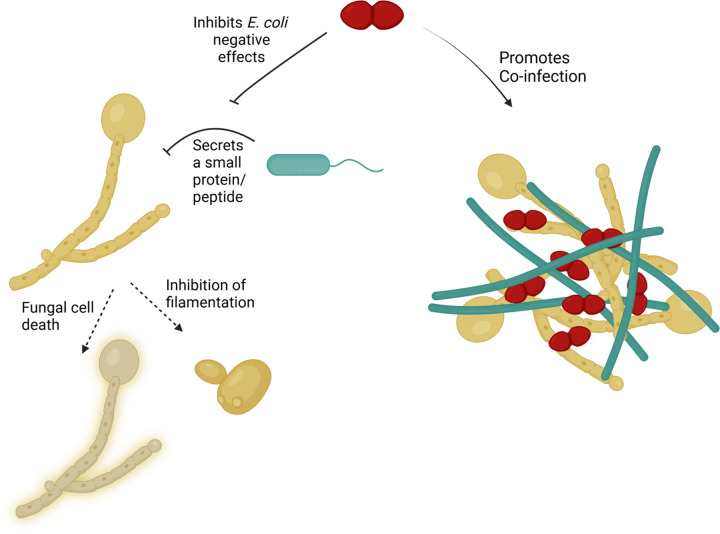
*C. albicans* occupy different niches in the bladder due to killing by *E. coli* but form mixed biofilms with *E. coli* and *E. faecalis*. Graphical representation of localization and colonization of *C. albicans* in the presence of *E. coli* and *E. faecalis + E. coli*. Created in biorender.com. Flores-Mireles, A. (2026) https://BioRender.com/4k41a25

In conclusion, CAUTI polymicrobial dynamics are highly environment-dependent and strictly governed by priority effects. While *E. coli* actively antagonizes *C. albicans*, *E. faecalis* acts as a crucial community mediator, neutralizing this antagonism to enable coexistence (Fig. 6). Given that *E. faecalis* is among the most frequently isolated pathogens in polymicrobial catheter infections, it likely functions as a keystone species that remodels the catheterized bladder environment to facilitate cooperative colonization. These complex interactions highlight the critical inadequacy of traditional monomicrobial models and standard diagnostic cultures. Ultimately, deciphering the mechanistic drivers of these communities provides a vital framework for developing novel, targeted therapeutics to combat recalcitrant polymicrobial CAUTI.

## MATERIALS AND METHODS

### Human urine collection

Human urine was collected and pooled from at least two healthy female donors between 20 and 40 years of age. Donors had no history of kidney disease, diabetes, or recent antibiotic treatment. Urine was sterilized using a 0.22 μm filter (Sigma-Aldrich) and pH adjusted to 6.0–6.5. When supplemented with BSA (VWR Life Sciences), urine was filter-sterilized again following BSA addition. All participants signed an informed consent form and protocols were approved by the local Internal Review Board at the University of Notre Dame under study #19-04-5273.

### Silicone disk preparation

Disks of UM-silicone (Nalgene 50 silicone tubing, Brand Products) were cut using an 8 mm leather hole punch. UM disks were washed three times in PBS and air dried. Disks were inserted into the bottom of 96-well plate wells (Fisher Scientific). Plates were UV sterilized for >30 min prior to use. Human Fg free from plasminogen and von Willebrand factor (Enzyme Research Laboratory #FB3) was diluted to 1 mg/mL in PBS. 200 μL of 1 mg/mL Fg was added to each well with silicone disks, sealed, and left overnight at 4°C.

### Microbial Strains and Growth Conditions

Microbe strains used in this paper include *E. faecalis*OG1RF strain, uropathogenic *E. coli* UTI89 strain, and fungal *C. albicans* SC5314 strain. Unless otherwise noted,*E. faecalis* and *C. albicans* were grown static for ~18 hr in 10 mL of BHI (Hardy Diagnostics C5143)supplemented with 66 μg/ml of rifampicin and 50 μg/ml fusidic acid and YPD supplemented with 400 μg/ml of kanamycin, respectively. *E. coli* was grown shaking for 5 hrs at 37°C in LB (MP Biomedicals) supplemented in 50 μg/ml of kanamycin then static in fresh LB for 24 hrs then, supplemented into fresh LB for an additional 24 hrs static (2 × 24 hrs). All cultures were washed in PBS (Sigma) three times and resuspended in PBS with OD_600_ of ~0.5 for *E. faecalis* and *E. coli* and 6.0 for *C. albicans*. For differential selection and growth of the pathogens, *C. albicans* was grown in YPD agar plates containing 400 μg/ml of kanamycin, a concentration that will exclude both *E. faecalis* and *E. coli* growth. *C. albicans* grows very well in YPD media due its acidic pH, and *C. albicans* growth is restricted on LB and BHI media. *E. faecalis* was selected on BHI plates supplemented with 66 μg/ml of rifampicin and 50 μg/ml fusidic acid; these antibiotic concentrations inhibit *E. coli* growth. *E. faecalis* growth is restrictive in YPD and LB media. Verification of the strains was further confirmed by their distinctive colony morphologies.

### Biofilm Assay

To assess the biofilm formation on silicone material covered with Fg, media was removed from the 96-well plates the following day. Urine containing microbes at a concentration of ~10^6^ CFU/mL was incubated with the silicone disks (in 96-well plates) for 24 or 48 hours at 37°C. At the end of the incubation period, the supernatants were aspirated and diluted for CFUs. The silicone disks were washed three times with PBS and transferred into borosilicate glass tubes filled with 1 mL of sterile PBS (VWR; Cat: 47729-576). Silicone disks were sonicated for 30 minutes and plated for CFUs.

### Planktonic Growth Curve

To assess the growth in planktonic cultures, round-bottom 96-well plates were used to incubate the cultures. Urine (with and without supplementations) containing microbes at a concentration of ~10^5^ CFU/mL were incubated for 6, 12, 24, and 48 hours at 37°C. Supplementations include Fg, serum, and Fg + serum. Samples were taken and plated at different time points for CFUs. The data were normalized by dividing the recovered CFUs by the initial inoculum, effectively scaling the starting value to 1 for all samples to ensure a proportional comparison of growth or survival.

### Visualization of Biofilms

Precut ultra-thin cast silicone sheets (SiMPore; Cat: GASKET-UT-50PK) were used to grow biofilms. Silicone sheets were cut into 1.5 × 1.5 cm squares and incubated with 1 mg/mL of Fg overnight. The following day, silicone sheets were incubated with urine containing microbes at a concentration of ~10^6^ CFU/mL for 48 hours. The sheets were washed three times with PBS, fixed with 10% neutralized, blocked, and stained using Sheep anti-Fg, Mouse anti-EbpABC (*Enterococcus*), Goat anti-*E. coli*, and/or Rabbit anti-*Candida* primary antibodies (Sigma) (1:1000) and secondary antibodies (Invitrogen) (1:5000).Coverslips were mounted on the silicone sheets using ProLong Gold antifade mountant (Thermofisher) and imaged using Zeiss inverted light microscope. Images were taken at different magnifications (10×, 20×, 40×, and 100×). Zen Pro and Fiji-ImageJ software were used to analyze the images^99^.

### Mice Handling and Husbandry

Mice used in this study were ~6-week-old female WT C57BL/6 mice purchased from the Jackson Laboratory. The University of Notre Dame Institutional Animal Care and Use Committee approved all mouse infections and procedures as part of protocol number 25-01-9009. All animal care was consistent with the Guide for the Care and Use of Laboratory Animals from the National Research Council.

### Mouse Infection Model

Mice were subjected to transurethral implantation of a silicone catheter and inoculated as previously described^100^. Briefly, mice were anesthetized by inhalation of isoflurane and implanted with a 6-mm-long silicone catheter (Braintree Scientific, SIL 025). Mice were infected immediately following catheter implantation with 50 μl of ~1 × 10^6^ CFUs/mL in PBS, of one, two, or three combinations of the microbial strains introduced into the bladder lumen by transurethral inoculation. Mice were euthanized at 1 dpi or 3 dpi by cervical dislocation after anesthesia inhalation, and the catheter, bladder, kidneys, spleen, and heart were aseptically harvested. Organs were homogenized, and catheters were cut into small pieces before sonication for CFU enumeration. Pathogens were plated in their corresponding media conditions (see Microbial strains and growth conditions). A subset of bladders was fixed and processed for immunohistochemistry staining and microscopy, histology analysis, and scanning electron microscopy (SEM) as described below.

### Immunohistochemistry Staining of Mouse Bladders

Mice bladders were fixed in 10% formalin overnight, before being processed for sectioning and staining as previously described^101^. Briefly, bladder sections were deparaffinized, rehydrated, and rinsed with water. Antigen retrieval was accomplished by boiling the samples in Na-citrate, washing in tap water, and then incubating in 1× PBS three times. Sections were then blocked [1% BSA and 0.3% Triton X-100 (Acros Organics, 21568-2500) in 1× PBS], washed in 1× PBS, and incubated with appropriate primary antibodies diluted in blocking buffer overnight at 4°C. Next, sections were washed with 1× PBS, incubated with secondary antibodies for 2 hours at room temperature, and washed once more in 1× PBS before Hoechst dye staining. Secondary antibodies for immunohistochemistry were Alexa Fluor 488 donkey anti-goat, Alexa Fluor 550 donkey anti-rabbit, and Alexa Fluor 650 donkey anti-rat. H&E stain for light microscopy was done by the core facilities at the University of Notre Dame (ND Integrated Imaging core). All imaging was done using a Zeiss inverted light microscope and a Leica Stellaris 8 DIVE ConfocalMicroscope. Zen Pro and ImageJ software were used to analyze the images.

### Scanning Electron Microscopy Sample Processing and Imaging

Mice bladders were fixed in 2% glutaraldehyde overnight and rinsed 3 times with buffer to remove excess glutaraldehyde. Bladders went through a secondary fixation of 1% osmium tetroxide and then washed for 10 mins with a buffer and rinsed 2 times with water to remove excess salts. Fixatives are diluted in buffer (0.1M PBS). Bladder samples were dehydrated in an ethanol series. Ethanol was added in 25% increments for 10 minutes each until it reached 100% (25%, 50%, 75%, 100%). Samples were washed 3 times with 100% ethanol and stored overnight in 25–75% ethanol. After dehydration steps, samples were put in the Critical Point Dryer (CPD). Once dried, samples were mounted on aluminum SEM stubs and coated with carbon. All SEM imaging was done using the Magellan 400 Field Emission Scanning Electron Microscope (FESEM) with extra high spatial resolution (XHR): 0.6 nm @ 15 kV, 0.9 nm @ 1 kV.

### Relative Hyphal Count using Imaging and qPCR

After biofilm assays were performed, some of the samples were used for CFUs and the rest of the samples were used for hyphae quantification. For the supernatant, the cultured urines were centrifuged for 1 minute at 14,800 rpm. The supernatants were removed, and the pellets were fixed with 10% formalin for 30 minutes. After 30 minutes, the samples were centrifuged, and the formalin was removed. Pellets were resuspended in 1x PBS and 10 **μ**L was mounted on a slide. Representative images were taken to count the ratio between yeast, pseudohyphae, and hyphae. For the biofilms, DNA was extracted from the sonicated samples using the Wizard^®^ Genomic DNA Purification Kit. *C. albicans* strain that is unable to form hyphae, *efg1*Δ/Δ, was used as a standard for the qPCR. ITS2 housekeeping gene was used as a reference for the quantification of cells. The 5’→3’ sequence of the primers are: CACACACACCTCTCCCTCAAA (ITS2 Forward) and TGAAGATATACGTGGTGGACG (ITS2 Reverse).

### Sequential Biofilm Assay

Silicone disks and microbial cultures were prepared as previously mentioned (see Silicone Disk Preparation and Biofilm Assay). Urine containing one of the microbes at a concentration of ~10^6^ CFU/mL was incubated with the silicone disks (in 96-well plates) for 24 hours at 37°C. The silicone disks were washed three times with sterilized 1× PBS and fresh urine containing microbe(s) at a concentration of ~10^6^ CFU/mL were added. Fresh urine with no bacteria/fungi was added the second day as a standard. The urine was incubated for another 24 hours. At the end of the incubation period, the supernatants were aspirated and diluted for CFUs. The silicone disks were washed three times with PBS and transferred into borosilicate glass tubes filled with 1 mL of sterile PBS (VWR; Cat: 47729-576). Silicone disks were sonicated for 30 minutes and plated for CFUs.

### High Inoculum Biofilm Assay

Silicone disks and microbial cultures were prepared as previously mentioned (see Silicone Disk Preparation and Biofilm Assay). Urine containing a combination of microbes at a concentration of ~10^7^ was incubated with the silicone disks (in 96-well plates) for 45 minutes at 37 °C. The silicone disks were washed three times with sterilized 1× PBS and fresh urine was added to each well. Silicone disks were incubated at different time points (0 hrs, 6 hrs, 12 hrs, 24 hrs, and 48 hrs) before supernatants were diluted for CFUs and silicone disks were washed, sonicated, and plated for CFUs.

### RNA-Sequencing

Biofilms were grown on 12-well plates (Non-Surface Treated Culture Plates; CELLTREAT^®^; Cat: CEL229512) that were covered with 1 mg/mL Fg overnight. Each well was filled with 3 mL of urine containing microbes at a concentration of ~10^6^ CFU/mL. The plate was incubated for 48 hours at 37°C. At 48 hours, supernatants were removed, and biofilms were washed once with 1× PBS. 800 **μ**L of TRI reagent (Zymo Research; Cat: R2050-1-200) was added to each well and cell scrapers (VWR; Cat: 10062-904) were used to collect the biofilms. RNA was extracted from the biofilms using Direct-zol RNA Miniprep kit (Zymo Research; Cat: R2053) and sent to GENEWIZ for sequencing. The results were analyzed on Galaxy (usegalaxy.org) (HISAT2, StringTie, HTseq, DESeq2). Venn diagrams were created using Evenn (ehbio.com) and Adobe Photoshop. Gene network was created using STRING (string-db.org) and further analyzed using Cystoscape. All statistical graphs were made in GraphPad Prism. The data discussed in this publication have been deposited in NCBI's Gene Expression Omnibus^102^ and are accessible through GEO Series accession number GSE329180 (https://www.ncbi.nlm.nih.gov/geo/query/acc.cgi?acc=GSE329180).

### Statistical Analysis and Rigor

Data derived from this study was entered into Graphpad Prism 9 to generate statistical results and graphs. Kolmogorov–Smirnov analytical tests and quantile-quantile (QQ) plot visual normality tests were performed to assess data distributions which, based on results, determined the appropriate parametric or non-parametric test to use. Normally distributed data was tested for difference between two groups with a student T-test, while an ANOVA followed by a Tukey’s post-hoc was used to determine statistical differences between more than two groups. For non-normal distributions, a Mann–Whitney U test was used to determine statistical difference between two groups, while a Kruskal–Wallis test followed by a Dunn’s test was used to determine significance between more than two groups. Pearson’s correlation statistical analysis was used to measure association between variables. Statistical tests used are indicated in figure legends.

## Supplementary Material

Supplementary Files

This is a list of supplementary files associated with this preprint. Click to download.

• ExtendedDataFigures042726.docx
